# Changes in surface characteristics and adsorption properties of 2,4,6-trichlorophenol following Fenton-like aging of biochar

**DOI:** 10.1038/s41598-021-82129-z

**Published:** 2021-02-22

**Authors:** Liqiang Cui, Qinya Fan, Jianxiong Sun, Guixiang Quan, Jinlong Yan, Kiran Hina, Hui Wang, Zhiqiang Zhang, Qaiser Hussain

**Affiliations:** 1 School of Environmental Science and Engineering, Yancheng Institute of Technology, No. 211 Jianjun East Road, Yancheng, 224051 China; 2 Department of Environmental Sciences, Hafiz Hayat Campus, University of Gujrat, Gujrat, 54000 Pakistan; 3 Institute of Soil Science, Pir Mehr Ali Shah Arid Agriculture University, Rawalpindi, 46300 Pakistan

**Keywords:** Environmental sciences, Solid Earth sciences

## Abstract

Fenton-like system formed in a natural soil environment deemed to be significant in the aging process of biochar. Aged biochars have distinct physico-chemical and surface properties compared to non-aged biochar. The aged biochar proved to be useful soil amendment due to its improved elements contents and surface properties. The biochar aging process resulted in increased surface area and pore volume, as well as carbon and oxygen-containing functional groups (such as C=O, –COOH, O–C=O etc.) on its surface, which were also associated with the adsorption behavior of 2,4,6-trichlorophenol (2,4,6-TCP). The biochar aging increased the adsorption capacity of 2,4,6-TCP, which was maximum at pH 3.0. The 2,4,6-TCP adsorption capacity of aged-bush biochar (ABB) and aged-peanut shell biochar (APB) was increased by 1.0–11.0% and 7.4–38.8%, respectively compared with bush biochar (BB) and peanut shell biochar (PB) at the same initial concentration of 2,4,6-TCP. All biochars had similar 2,4,6-TCP desorption rates ranging from 33.2 to 73.3% at different sorption temperatures and times. The desorbed components were mainly 2,4,6-TCP and other degraded components, which were low in concentration with small molecule substance. The results indicated that the aged-biochar could be effective for the long-term remediation of naturally organic polluted soils.

## Introduction

Trace organic compounds act as pollutants in waste water and pose a major threat to the human health and ecosystem security/safety^1^. Organic pollution is usually affected by the physical and chemical properties of the sorbent on which sorption–desorption reactions occur. Biochar properties (such as surface characteristics, aging, etc.) affect the infiltration of wastewater into soils which are crucial for protecting water resources and minimizing health hazards^2^.

Biochar is carbonaceous and porous material which is produced from waste biomass through pyrolysis technique^3^. Because of the specific surface characteristics (porous structure, micropores etc.), abundant microchannels and stable aromatic chemical structure (such as alkaline pH and rich organic functional groups)^4^, biochar has been widely used as an environmentally friendly amendment for the treatment of wastewater and soil pollution^5^. For example, wood chip biochar (3–5%, wt) had a high ability to remove 2,4‐dinitrotoluene (DNT) and 2,4‐dichlorophenol (DCP) from an aqueous solution^6^.

In the environment, biochar interacts with environmental constituents or factors and undergoes a series of biogeochemical reactions (i.e. aging process) that result in alteration of the biochar’s surface properties over time^7^. The biochar has ability to decrease organic pollutant’s bio-availability and transport through increased sorption and/or degradation^8^. The sorption strength of organic pollutants depends on physical and chemical properties of the biochar, which vary between different feedstock materials and aging time durations in amended soils^9,10^. Increasing adsorption capacity with aged biochar would decrease the available organic pollutants concentration, thereby limiting their activity and promoting biodegradation over time^2^. Biochar also decreases degradation of organic pollutants such as pesticides in soils, often attributed to increased pesticide sorption and decreased pesticide bioavailability. For example, Loganathan et al.^11^ found that biochar amended soils contained higher non-desorbable fractions of atrazine compared with control which contained more rapidly mineralizing atrazine.

The biochar undergoes physical and chemical changes over time in soils, as a result of which its pore structure and particle size are changed, influencing its recalcitrant and pollutants sorption ability^12^. Biochar in soil is oxidized with aging as it acts primarily as a reducing agent over a long period of time. In order to accelerate aging process, the simulating experiments had been done in the laboratory to assess the aging effects on the biochar properties^13^. The nano-iron/nickel modified biochar effectively removed up to 71.6% of 2,4,6-trichlorophenol (2,4,6-TCP) from the natural waste water treatment^14^. Zhuang et al.^15^ also reported that Fe_3_O_4_ activated-hyacinth biochar removed up to 98.9% of 2,4,6-trichlorophenol from waste water by enhancing fermentation performance. Biochar aging is also a natural process that alters biochar properties to immobilize the heavy metals pollutants in different environment^13^. In the natural aging process, wheat straw biochar significantly reduced bioavailability of heavy metals concentration in paddy soils^16^. The 12-month aging of the biochar increased the Cd adsorption capacity in the soil^17^.

Abiotic processes were more important than biotic processes for the initial oxidation of fresh biochar, which was used to simulate the natural aging process in the laboratory experiment^18^. The modified poplar catkins biochar exhibited maximum adsorption capacities of 384 mg g^−1^ for chloramphenicol in wastewater^19^. Moreover, Fenton-like oxidizing agents (H_2_O_2_ and NaClO) modified biochar showed a high ability to sorb phenanthrene in waste water^20^.

The governing mechanisms for biochar in sorbing of organic pollutant are its high external and internal surface area and oxygen-containing (e.g. carboxyl and hydroxyl) surface functional groups, which enhance the biochar’s sorption ability to organic pollutants by promoting π–π and electrostatic interactions, especially for the aging biochar^21^. To protect soil and surface waters from contamination, knowledge of organic pollutants degradation and sorption–desorption processes is necessary. During plant nutrient uptake, root sorption governs the bioavailability of organic pollutant such as 2,4,6-TCP to plant in soil solution. It is well documented that the biochar has a greater ability to sorb the organic pollutant, but mechanisms of the aged biochar, its properties and sorption of the organic pollutant are less explored. Moreover, there is a limited research on the simulated aging of biochar and role of biochar’s surface characteristics when it is applied to polluted soil. So, we hypothesize that surface properties and acidic functional groups of the biochar improve with the aging process, which in turn increases sorption ability of the organic pollutant onto biochar. Therefore, in this study, aging of biochar, derived from peanut shell and bush, was simulated by chemical oxidation with citric acid/FeCl_3_ and citric acid/α-Fe_2_O_3_ systems. In an effort to investigate the impact of aging treatment on the surface chemistry of biochar and the associated adsorption capacity and sorption–desorption processes, we try to understand the aging mechanisms in the Fenton-like system that occur in the natural soil environment.

## Materials and methods

### Biochar and 2,4,6-TCP preparation

The 2,4,6-TCP (> 98%) was provided by Jiangsu Academy of Agricultural Sciences. A 2,4,6-TCP stock solution (500 mg L^−1^) was prepared by dissolving 2,4,6-TCP in methanol, followed by storage at 4 °C in the dark to avoid photo-chemical degradation.

The peanut shell (collected from farmers) and bush (roadside green belt plant of school) were air dried and crushed into small pieces, and then further dried in the oven at 105 °C. The dried peanut shell and bush materials were pyrolyzed at 450 °C under anaerobic conditions in the vacuum tube furnace (NBD-O1200, Nobody Materials Science and Technology CO., LTD, Zhengzhou, China) and the prepared biochars were dried at 105 °C for further experimental use in the laboratory^9^. The biochar was sieved to pass through a 2 mm mesh and analyzed for several chemical and physical properties (Table S1).

### Simulated chemical aging

Chemical aging was conducted to simulate different natural field conditions. For this purpose, the citric acid/FeCl_3_ and citric acid/α-Fe_2_O_3_ mixtures were chosen to oxidize peanut biochar and bush biochar as mineralization models of biochars in acidic soil.

The Fenton-like aging process was initiated by immersing 05 g biochar in 100 mL 0.30 mM L^−1^ Fe^3+^ (*α*-Fe_2_O_3_, Shanghai, China) solution, and then adding 400 mL of 0.01 M buffer solution (Citric acid/sodium citrate, pH 4), after that UV irradiation was applied by a photochemical reaction apparatus (Xujiang electromechanical plant, Nanjing, China) with 500 W high pressure Hg lamp for 6 h^13^. The biochar was then separated with 0.45 μm glass filter paper (Whatman #934-AH). To remove residual solution, aged biochars were washed repeatedly with deionized water for 1 h until pH of the filtrate was stabilized. The aged biochar was dried in the air-circulating oven at 105 °C for 4 h. All treatments were run in triplicate.

### Chemical characterization

#### Elemental analysis

The C, H, N and S contents were analyzed by Vario EL Cube instrument (Elementar, Germany). Oxygen content was calculated as the difference between the sum of C, H, N, S and ash mass fractions^13^.

#### Fourier transform infrared spectroscopy (FTIR)

The FTIR spectra were recorded in the region of 400–4000 cm^−1^ by using a Waltham Nexus-670 FTIR Spectrometer (NEXUS-670, NICOLET, USA) with a resolution of 1.0 cm. The biochar pellets were prepared by using KBr powder.

#### X-ray photoelectron spectroscopy (XPS)

XPS was used to analyze surface properties in approximately 10 nm depth of biochars. XPS measurements were conducted using an ESCALAB 250Xi (THERMO FISHER, USA), equipped with a focused monochromatized Al Kα radiation (hν = 1486.6 eV). The X-ray spot size was 500 μm. Survey scan spectra in the 1351–0 eV binding energy range were recorded with a pass energy of 100.0 eV, others in 20.0 eV^13^. The spectra were separated using Systat Peakfit, Version 4.12 (Seasolve, China)^5^.

#### Scanning electron microscope: energy dispersive spectrometer (SEM–EDS)

Scanning electron microscope with energy dispersive spectroscopy (SEM–EDS) was carried out on Nova NanoSEM 450 (FEI, USA) and AZtec X-MaxN 80 (Oxford, UK). SEM was operated at 15 kV with a probe current of 0.6 nA to which a Bruker silicon drift detector EDS system had been interfaced^16^.

### Batch adsorption and desorption experiments

A series of batch sorption/desorption experiments were performed in triplicates to evaluate the 2,4,6-TCP adsorption/desorption capacity of aged biochars. For the adsorption experiment, different 2,4,6-TCP concentrations, ranging from 5 to 160 mg L^−1^ were prepared from the stock solution. The 2,4,6-TCP solution and biochars were mixed in a 100 mL glass flask, and sealed with tape and shaken at 180 rpm for 2 h in a water bath at 25 °C, 35 °C, and 45 °C. The background electrolyte of the mixed solution was 0.01 mol L^−1^ NaNO_3_, and the pH was adjusted with 0.1 M NaOH or HCl^22^. After reaching the equilibrium during the sorption experiment, the solution was filtered through 0.22 μm nylon membrane. The 2,4,6-TCP concentrations were analyzed by high performance liquid chromatography (HPLC, Perkin Elmer Flexar-15, PerkinElmer Inc., Waltham, USA) with a UV detector equipped with a reverse phase column, 4.6 mm × 150 mm XDB-C18 column (Agilent Technologies Inc., Santa Clara, USA)^9^.

The adsorption capacity of 2,4,6-TCP was calculated according to the following Freundlich sorption isotherm equations:

$$ {\text{lgQe }} = {\text{ lgK}}_{{\text{F}}} + {1}/{\text{n lgCe}} $$

where: Ce (mg L^−1^) is the equilibrium liquid-phase concentrations of 2,4,6-TCP, Qe (mg g^−1^) is the amount of 2,4,6-TCP adsorbed per gram of biochar at the equilibrium state, and Kf (g mg^−1^) are rate constants for Freundlich sorption isotherm model, 1/n is the Freundlich constant related to the surface heterogeneity.

The biochars adsorbed with 2,4,6-TCP were washed with distilled water three times to remove the surface 2,4,6-TCP, followed by addition of 50 mL 0.01 mol L^−1^ NaNO_3_ to desorb the 2,4,6-TCP from the biochars and shaken for 2 h at 25 °C. After shaking the solution was filtered, and the 2,4,6-TCP concentration in the filtrate was measured as described above. At the end of the desorption experiment, parts of the solution were adjusted for the pH (< 2 with HCl) and extracted with 50 mL dichloromethane (chromatographically pure) and was dried with anhydrous sodium sulfate. The extract was analyzed for 2,4,6-TCP and the degradation products using Thermo Trace DSQ II Gas Chromatography-Mass Spectrometry (GC–MS) (TRACE 1310- ISQ, Thermo Fisher Scientific, Waltham, USA).

### Statistical analysis

All data were expressed as means ± one standard deviation. Differences between the treatments were examined using a two-way analysis of variance (ANOVA), with statistical differences considered when *P* < 0.05. All statistical analyses were carried out using SPSS, version 20.0 (SPSS Institute, USA).

## Results and discussion

### Compositional changes

The surface properties of biochars were analyzed by XPS with a low penetration depth of X-rays (Table 1). The relative proportion of carbon (C) was increased in pristine biochar as compared to aged biochar, which was probably due to the inorganic carbon being dissolved in acid solution during aging processing. The C contents of BB and ABB were increased from 67.2 to 68.57% and 55.8 to 58.5% for PB and APB, respectively. The N contents showed significant differences in bulk element and surface composition, which could be due to the effects of aging process on the biochar surface as described by Fan et al.^13^. The aged biochars surface sulfur was increased by 12.7% (ABB) and 26.9% (APB) compared to BB and PB, respectively. The percentage of ash content in aged biochars decreased to 28.32% (ABB) and 21.56% (APB) compared to the control, respectively (Table 1).

**Table 1:** Characterization of biochars.

Samples	Bulk element composition (%)^a^			Surface composition (%)^b^				
	N	C	Ash	N	C	O	S	pH
BB	0.9	67.2	30.20	3.06	82.04	12.42	0.63	9.51
ABB	1.07	68.57	28.32	2.9	82.62	11.12	0.71	/
PB	1.05	55.8	23.12	2.8	79.77	14.39	0.67	9.16
APB	1.48	58.5	21.56	2.67	77.1	16.01	0.85	/

*BB* bush biochar, *ABB* aged bush biochar, *PB* peanut shell biochar, *APB* aged peanut shell biochar.

^a^Data obtained using an elemental analyzer.

^b^XPS measurement.

Significant differences were found in different parameters of bulk and surface chemical composition (Table 1). The relative proportion of C and N was higher in surface than that in bulk elemental composition of aged biochar, such differences could be due to the different interior and the surface structure of biochar. The surface layer consisted of persistent carbon that is formed by degradable carbon which is broken down/dissolved during the aging process^23,24^. Therefore, aging process played an important role in remodeling the properties of biochar, which could generate –OH as in the Fenton-like system, which probably promoted the degradation of organic pollutant.

Aged biochar is developed due to changes in surface chemical characteristics, carbon loss and other elements incorporation that occur during whole aging process. The significant variations between different surface parameters and the bulk composition showed that biochar was equipped with the aging layer consisted of persistent carbon, along with high C and N ratio, on the surface.

### Functional groups of biochars

**Figure 1:**
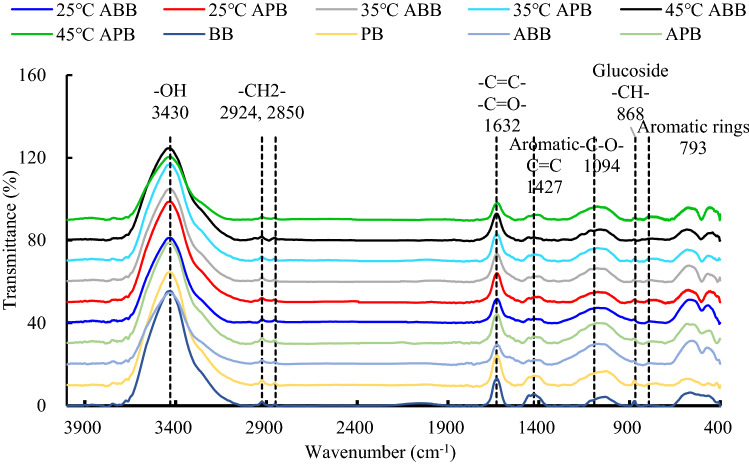
Infrared spectra of biochars from different process (BB, PB, ABB, APB without temperatures mean the biochars no sorbing 2,4,6-TCP; BB, PB, ABB, APB with different temperatures mean the biochars sorbing 2,4,6-TCP at different temperatures).

The FTIR (Fourier transform infrared spectroscopy) was conducted to detect the functional groups in the biochars as shown in Fig. 1. The functional groups for band assignments of FTIR are provided in Table 2. The broad spectrum band at 3400 cm^−1^ was the result of –OH stretching vibration (ν_O–H_), which could be due to Fenton-like system agents incorporation onto the surface of the biochars, that was also confirmed by other studies^24,25^. Peaks at 2950–2860 cm^−1^ were the stretching of –CH_2_– or –CH_3_ in aliphatics or alicyclics (ν_C–H_) and showed a slight decrease in aged biochar after sorption of the 2,4,6-TCP (Fig. 1). The peak at 1632 cm^−1^ represented stretching of C=O groups that were connected with the aromatic ring, or –COOH (ν_C=O_), and showed a transmittance decrease in sorbed biochar, which indicated the decrease of carboxyl group. A decrease of stretching appeared in aromatic C–O (ν_C–O_) at 1094 cm^−1^. Aliphatic CH_2_ deformation (868 cm^−1^) may be replaced by 2,4,6-TCP, which was decreased after sorption (Fig. 1). The struvite crystallization, electrostatic attraction, and π–π interactions of the aged biochar played key roles to remove the dissolved organic matter (e.g. humate) in the wastewater (swine factory)^8,26^. Mia et al.^20^ also proved that the functional groups of aged biochars play important roles in interacting with the organic chloride and increasing degradation.

**Table 2:** Band assignments in FTIR spectra of biochar.

Bands (cm^−1^)	Assignments	References
3400–3320	–OH stretching	Chen et al.^25^; Hu et al.^24^
3000–2800	Aliphatic CH stretching	Cui et al.^22^
1630–1700	Aromatic carbonyl/carboxyl C=O/C=C stretching	Cimo et al.^27^
1430–1420	Aromatic C=C stretching	Cui et al.^9^
1000–1157	C–O–C	Fan et al.^13^
840–880	Glucoside CH_2_ deformation	Mia et al.^20^
750–820	Aromatic rings	Li et al.^26^

**Figure 2:**
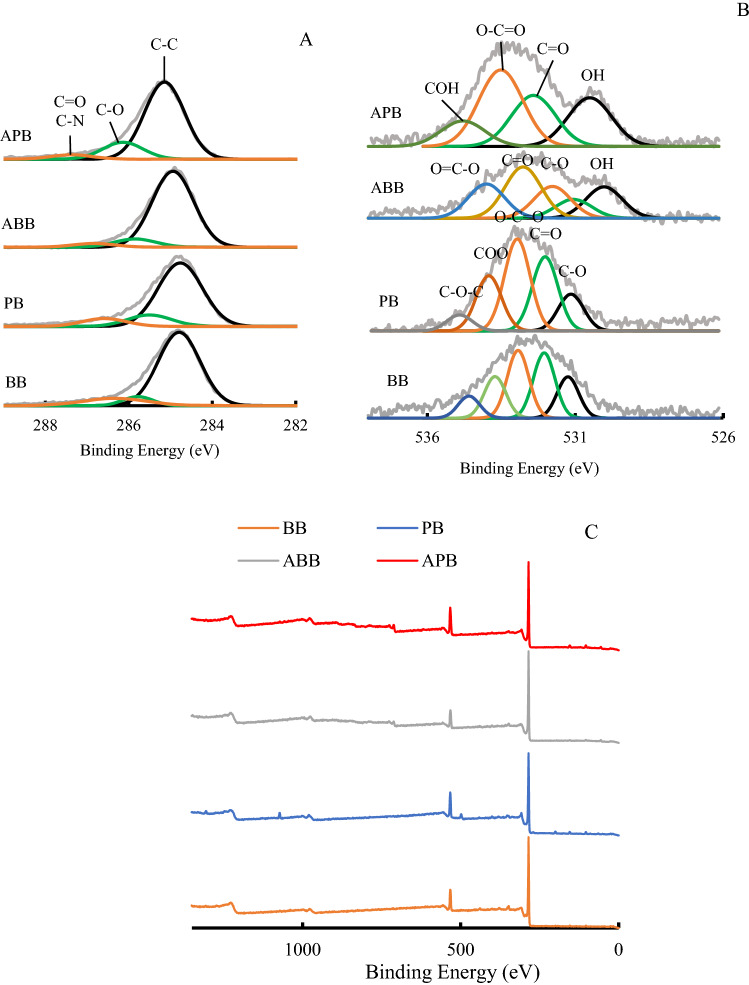
Peak fits of C1*s* (**A**), O1*s* (**B**) and maps (**C**) from different biochars.

Further analyses of C and O were conducted by using XPS technique (Fig. 2). Data from XPS showed a weakening in C and strengthening in O after aging process, so peak-fit on C1*s* and O1*s* was done to check the changes (Fig. 2C). Three peaks were found in C1*s*, i.e. C–C (284.8 eV), C–O (286.1 eV), C=O (287.0 eV). The main four peaks for O1*s* were C=O at 532.5 eV, O–H at 530.3 eV, O–C=O at 532.8 eV, and C–OO at 533.8 eV. The O–H functional group was obviously found in aged biochar, which was less pronounced in the fresh biochar. Aliphatic stretching was found to decrease in O1*s*, the increased O groups was probably focused on aromatics^13^. Aging biochars showed adsorption peaks of C−O, C=O and O−C=O bonds due to surface oxidation. As a result of rapid oxidation of the surface of the biochar, excessive free radicals are formed at the earlier stage of aging, which initially leads to the formation of C–O and O–H bonds, and later some of them are converted to C=O and O–C=O as the aging process continues. During the aging of biochars, the proportion of aliphatic C−H increased relative to the aromatic C−H, this may be due to the opening of the benzene ring or the volatilization of small molecules containing the benzene ring^28^. The FTIR and XPS results showed that aged biochars were equipped with more oxygen-containing functional groups^27^. The singlet oxygen (^1^O_2_) was the main reactive species for organic pollution degradation, which was confirmed in the modified biochar for methylene blue sorption^29^.

In brief, both FTIR and XPS showed the increase of C and O throughout the aging process, which was in accordance with the compositional analysis. This may be the result of ash, carbonate and few dissolvable carbons leaching which washed down or decomposed into inorganics, while persistent C like aromatics remained intact. The increased O was noticed in oxygen-containing functional groups in aromatic C structure from XPS and oxidation reaction, especially carboxyl aromatics.

### Surface characteristics and the minerals

**Figure 3:**
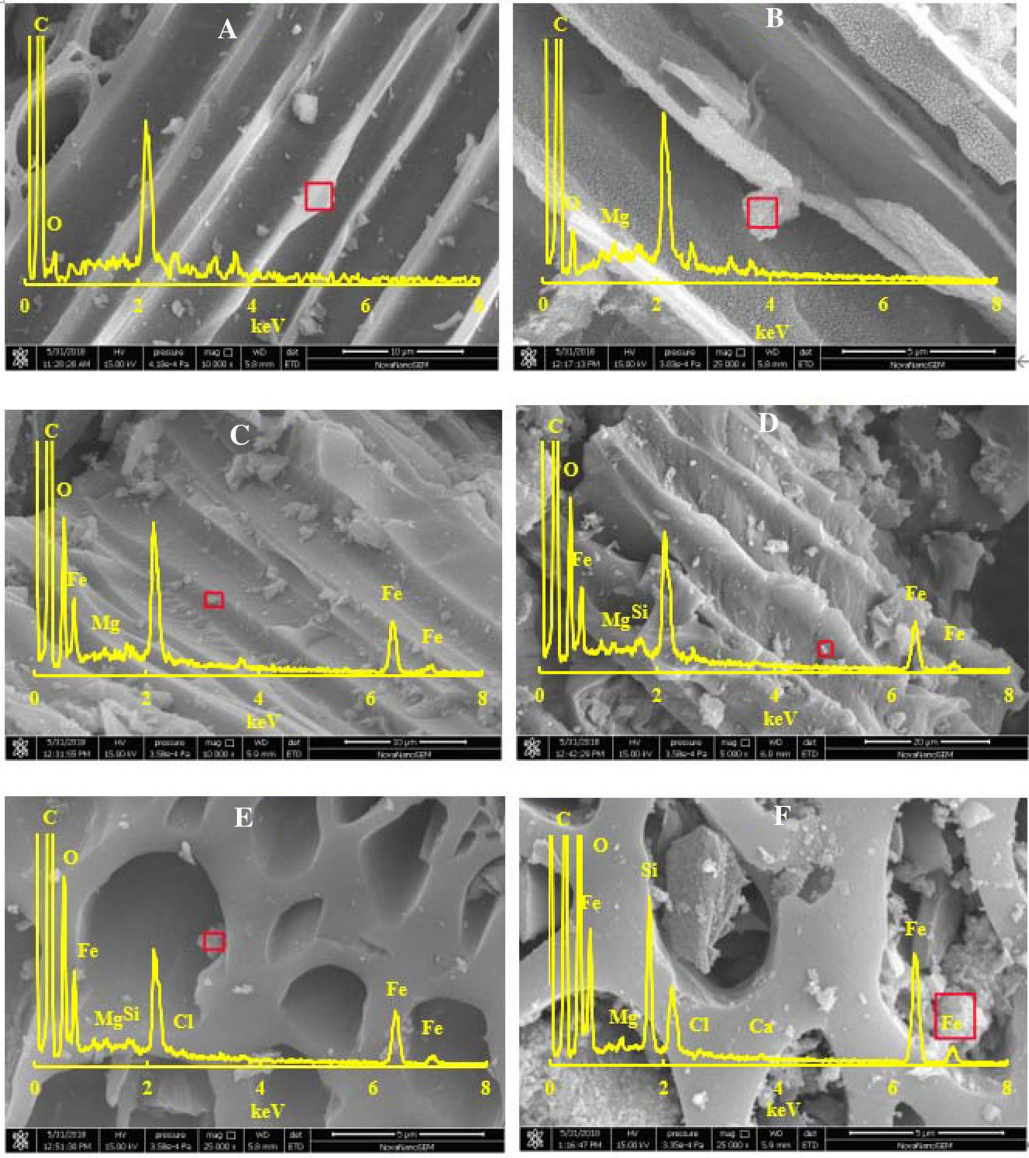
The SEM and EDS of BB (**A**), PB (**B**), ABB (**C**), APB (**D**), ABB sorbed 160 mg L^−1^ 2,4,6-TCP (**E**) and APB sorbed 160 mg L^−1^ 2,4,6-TCP (**F**).

Surface morphology of biochars are shown in Fig. 3. The SEM–EDS analysis showed smooth (Fig. 3A,B), crude surface and precipitate points (Fig. 3C–F) on biochars with and without sorbed 2,4,6-TCP (Figs. S1–S4). The structures of biochars were corroded by Fenton-like system after the aging process. The aged biochars showed higher Fe ratio compared with fresh biochars (Fig. 3E,F). More wrinkles or cracks in lengthways were generated on the surface during aging process, thus the surface of biochar creates more active sites, which facilitates the adsorption of 2,4,6-TCP. With 2,4,6-TCP sorption, the Cl element was shown on the surface, then the 2,4,6-TCP probably formed compound with biochar surface functional groups. Iron based minerals were also found to be increased rapidly on the biochar surface, especially for aged biochar. Moreover, minerals were likely infused into pores of biochar and formed mineral nanostructures^30^. So, with aging the surface area and pore volume of biochars were also increased by 29.1% (APB), 23.5% (ABB) and 43.4% (APB), 32.4% (ABB) compared with control (Table S2). The contents of non-protonated aromatic carbon, micropore volumes, and micropore sizes are the critical factors to micropore filling mechanism of organic pollutants onto biochar^24^.

### The effects of pH on 2,4,6-TCP adsorption

**Figure 4:**
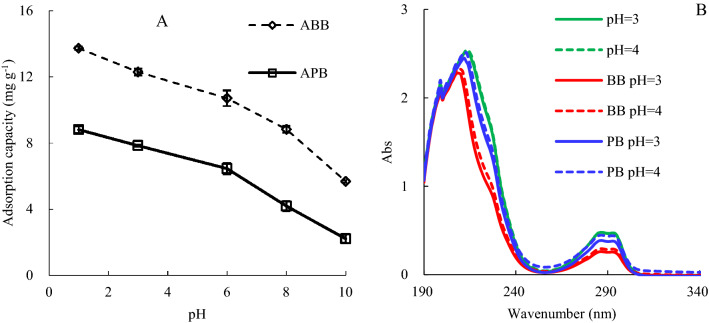
Effects of initial pH on 2,4,6-TCP adsorption (**A**) the biochars adsorption capacity with different solution pH; (**B**) Abs of band ultraviolet scanning with different 2,4,6-TCP solution pH.

Initial solution pH has greater effects on biochar sorption of 2,4,6-TCP (Fig. 4). At a relatively low pH, hydrogen ions were easy to be unionized from 2,4,6-TCP, because there was already plenty of H^+^ in the solution, so 2,4,6-TCP was easier to be sorbed on biochar compared to ionized 2,4,6-TCP with negative charge (e.g. lost the H^+^). When the solution pH was lower, the biochar adsorption capacity was higher, therefore, the better adsorption was at low pH in this study (Fig. 4A). The adsorption capacity increased with the aged biochar, so kinetic and adsorption isotherms experiments were conducted to study the mechanism of 2,4,6-TCP adsorption by biochars. The solution pH was adjusted to 3 after completion of sorption study, so the 2,4,6-TCP was kept at molecular state and at the peak point (Fig. 4B).

### Adsorption kinetics and isotherms

The Freundlich sorption isotherm was fitted to study the effects of initial 2,4,6-TCP concentration on maximum adsorption capacity of biochars (Table 3). The Freundlich sorption isotherm did fit very well as indicated by high correlation coefficients (*R*^2^).

**Table 3:** Parameters of Freundlich isotherm on 2,4,6-TCP adsorption.

Temperature		*n*	*K*_F_	*R*^2^	p	Equation
25 °C	BB	2.25	5.23	0.9874	0.0006	lgQe = 0.72 + 0.44 × lgCe
	PB	0.66	0.03	0.9405	0.0063	lgQe = − 1.50 + 1.51 × lgCe
	ABB	2.58	7.02	0.9836	0.0009	lgQe = 0.85 + 0.39 × lgCe
	APB	0.61	0.02	0.9083	0.0121	lgQe = − 1.67 + 1.64 × lgCe
35 °C	BB	3.41	10.55	0.9448	0.0056	lgQe = 1.02 + 0.29 × lgCe
	PB	0.84	0.17	0.9125	0.0113	lgQe = − 0.77 + 1.18 × lgCe
	ABB	3.77	11.90	0.9082	0.0121	lgQe = 1.08 + 0.27 × lgCe
	APB	1.12	0.70	0.9225	0.0094	lgQe = − 0.16 + 0.89 × lgCe
45 °C	BB	4.29	13.42	0.8968	0.0145	lgQe = 1.13 + 0.23 × lgCe
	PB	1.07	0.55	0.9689	0.0023	lgQe = − 0.26 + 0.94 × lgCe
	ABB	5.06	15.75	0.8281	0.0320	lgQe = 1.20 + 0.20 × lgCe
	APB	1.15	0.88	0.8486	0.0262	lgQe = − 0.05 + 0.87 × lgCe

**Figure 5:**
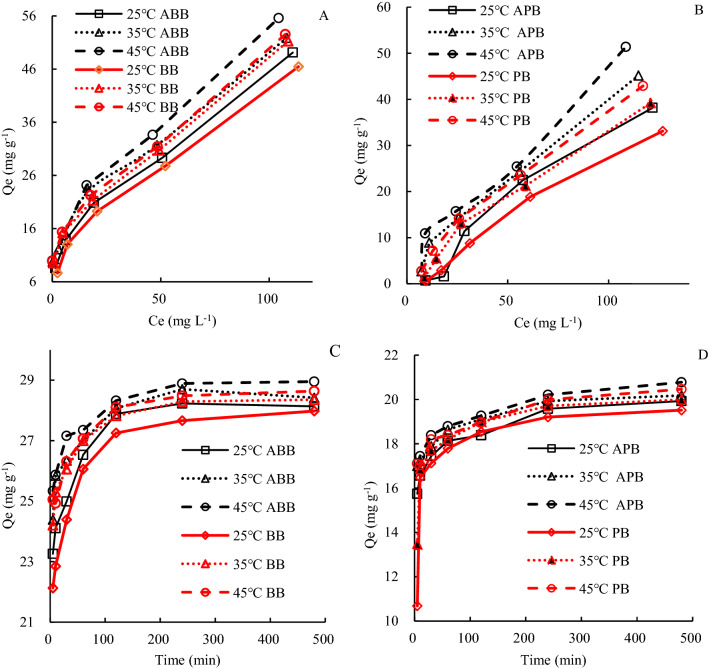
Adsorption with different initial concentration [(**A**) Bush biochar; (**B**) Peanut biochar)] and time [(**C**) Bush biochar; (**D**) Peanut biochar)] with different solution temperature.

The adsorption capacity of fresh biochars and aged biochars increased rapidly with the increase in initial concentration of 2,4,6-TCP and then tended to keep stable with time longer than 120 min and biochar reaching the saturation (Fig. 5). Aged biochar showed higher adsorption capacity at the same concentration or time when compared with fresh biochar. Adsorption capacity of ABB and APB increased by 1.0–11.0% and 7.4–38.8% compared to BB and PB, respectively. Kinetics study also showed a similar trend that adsorption increased slightly in 240 min. The fast sorption phase was caused by the solution diffusion of adsorbates and exterior surface adsorption onto biochars^13^. Over 240 min, sorption was increased at a low rate with a fluctuating trend, as the exterior surface was hard to adsorb more ions while the interior sorption was still developing. The adsorption performance of aged biochars was superior to fresh biochar with higher adsorption rate. Different adsorption rates indicated that adsorption of 2,4,6-TCP onto biochars was not only a physical reaction, but also a chemical reaction, which was confirmed in the 2,4,6-TCP desorption and GC–MS maps (Fig. 7).

### The 2,4,6-TCP desorption from biochars

**Figure 6:**
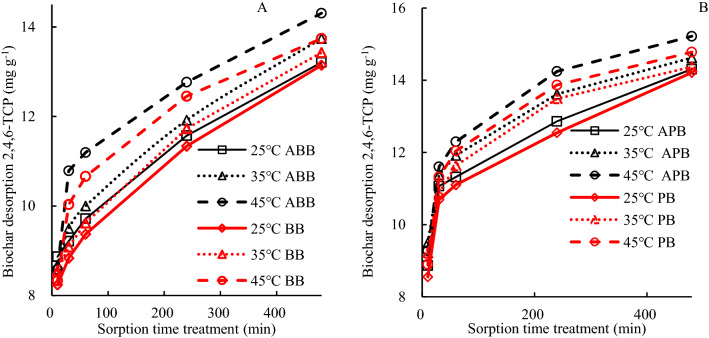
The biochar desorbing 2,4,6-TCP at different time and temperature (**A**) ABB and BB, (**B**) APB and PB.

The 2,4,6-TCP adsorbed on the biochar was also desorbed with desorption solution (Fig. 6, Table S3). The 2,4,6-TCP desorption ratio ranged from 33.2 to 49.4% (ABB), 53.2 to 73.3% (APB), 33.4 to 48.0% (BB), and 51.9 to 72.4% (PB) at 25 °C, 35 °C and 45 °C. The 2,4,6-TCP desorption concentration from biochars was increased with high solution temperature and longer time duration. The 2,4,6-TCP loaded on fresh or aged biochars was probably physical adsorbed, so it was easily eluted down. The aged (ABB) or fresh bush biochars (BB) more firmly adsorbed 2,4,6-TCP which was difficult to remove than aged (APB) or fresh peanut biochars (PB). The desorption rate of 2,4,6-TCP was related to the properties of the biochars derived from different feedstock.

**Figure 7:**
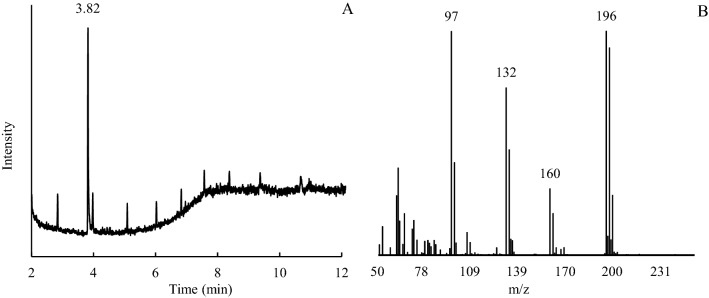
The GC–MS maps of desorbing 2,4,6-TCP from aging bush biochars in the solution (**A**) GC map, (**B**) MS map.

The 2,4,6-TCP degradation components, at the end of the degradation experiment, were analyzed by GC–MS (Fig. 7). The 2,4,6-TCP was detected mainly as 2,4,6-TCP (3.82 min; Fig. 7A), even after the biochar sorbed and desorbed again. In the desorption solution, other degradation components were a combination of low molecular weight organic matter which were low in concentration, they might come from 2,4,6-TCP degradation or biochar releasing (Fig. 7B).

The efficient adsorption of 2,4,6-TCP onto aged biochar was attributed to the improvement in the biochar surface properties and functional groups^31^. The micropore filling was the dominating adsorption mechanism; the micropore size distribution affected the adsorption mechanism of organic pollutants sorption. Hu et al.^24^ found that adsorption was the dominant sorption mechanism of phenanthrene by the oxidized biochars. The surface properties changed as increased oxygen-containing functional groups provided more adsorption sites, resulting in strong complexation of 2,4,6-TCP with aged biochars. The aging process played an active role in the 2,4,6-TCP sorption onto the biochars. So, the application of biochar in the wastewater could be a wise strategy for the remediation of organic contaminated water, which may increase the adsorption capacity over a longer period of time during natural aging process. Moreover, the disinfectant byproducts during chlorination promoted degradation to low molecular weight organic matter along with more active sites on the wheat straw biochar^32^. Kim and Hyun^33^ also found that the organic pollutions sorbed by *Miscanthus* derived biochar was greatly attributed to the effect of π–π electron donor–acceptor interaction and calcium complexation. In short, it can be concluded from current study that the sorption of 2,4,6-TCP was mainly determined by the special biochars properties and microstructure that included electron interaction and the formation of carbon bonds.

## Conclusion

Fresh and aged biochars have significantly different physico-chemical and surface properties. Aged biochars have better ability to sorb 2,4,6-TCP efficiently compared to fresh biochars. Adsorption of 2,4,6-TCP onto aged biochars was influenced by solution pH, contact time, temperature, biochar’s properties and strong interaction with active sites. The biochars sorption abilities for the 2,4,6-TCP were also influenced by types of feedstock. The mechanism of 2,4,6-TCP adsorption on the aged biochar was attributed to the acidic functional groups, amorphous carbon, and the complex micropore structure, which were different in aged biochar compared to fresh biochars. So, biochars could be used in the natural environment to remediate organic pollutants in wastewater for a longer time, as biochars with aging become more active and efficient.

## Supplementary information

Supplementary information.
