# Supplementary material for: Changes in surface characteristics and adsorption properties of 2,4,6-trichlorophenol following Fenton-like aging of biochar
Source: Sci Rep. 2021 Feb 22;11:4293. doi: 10.1038/s41598-021-82129-z (PMC7900105; doi:10.1038/s41598-021-82129-z)
Supplement: Supplementary file 1 — Supplementary information. [file 41598_2021_82129_MOESM1_ESM.docx]

Supplementary materials

**Changes in surface characteristics and adsorption properties of 2,4,6-trichlorophenol following Fenton-like aging of biochar**

Liqiang Cui^a^, Qinya Fan^a^, Jianxiong Sun ^a^, Guixiang Quan^a^, Jinlong Yan^a*^, Kiran Hina^b^, Hui Wang^a^, Zhiqiang Zhang ^a^, Qaiser Hussain^c^

a: School of Environmental Science and Engineering, Yancheng Institute of Technology, No. 211 Jianjun East Road, Yancheng 224051, China.

b: Department of Environmental Sciences, Hafiz Hayat Campus, University of Gujrat, Gujrat, 54000, Pakistan.

c: Institute of Soil Science, Pir Mehr Ali Shah Arid Agriculture University, Rawalpindi, 46300, Pakistan.

**Table S1** Basic properties of raw biochars

|  | pH  (H_2_O) | Organic carbon  (g kg^-1^) | CEC  (cmol kg^-1^) | Total N  (g kg^-1^) | Total P  (g kg^-1^) | Total K  (g kg^-1^) | Total Fe  (g kg^-1^) |
| --- | --- | --- | --- | --- | --- | --- | --- |
| PB | 9.16 | 558.2 | 9.54 | 10.52 | 44.73 | 15.51 | 2.11 |
| BB | 9.51 | 671.5 | 14.63 | 8.96 | 33.25 | 18.06 | 3.05 |

BB: bush biochar; PB: peanut shell biochar

**Table S****2** Surface area and pore volume of biochars

| Samples | PB | BB | APB | ABB |
| --- | --- | --- | --- | --- |
| Surface Area (m^2^ g^-1^) | 9.54 | 18.01 | 12.32 | 22.25 |
| Pore Volume (cm^3^ g^-1^) | 0.0325 | 0.0478 | 0.0466 | 0.0633 |

**Table S3** Biochar desorption 2,4,6-TCP capacities (mg g^-1^)

| Temperature (℃) | Time (min) | BB | PB | ABB | APB |
| --- | --- | --- | --- | --- | --- |
| 25 | 10 | 8.24±0.42 | 8.55±0.12 | 8.87±0.15 | 8.87±0.15 |
|  | 30 | 8.84±0.12 | 10.69±0.09 | 9.21±0.31 | 11.07±0.27 |
|  | 60 | 9.37±0.04 | 11.10±0.12 | 9.72±0.28 | 11.32±0.48 |
|  | 240 | 11.34±0.10 | 12.55±0.08 | 11.57±0.24 | 12.86±0.19 |
|  | 480 | 13.14±0.12 | 14.21±0.12 | 13.21±0.35 | 14.31±0.12 |
| 35 | 10 | 8.55±0.19 | 9.21±0.38 | 8.68±0.31 | 9.50±0.32 |
|  | 30 | 9.09±0.16 | 10.88±0.16 | 9.50±0.24 | 11.38±0.29 |
|  | 60 | 9.62±0.13 | 11.64±0.12 | 10.00±0.20 | 11.92±0.04 |
|  | 240 | 11.73±0.12 | 13.49±0.08 | 11.92±0.16 | 13.62±0.12 |
|  | 480 | 13.43±0.12 | 14.37±0.24 | 13.74±0.12 | 14.62±0.08 |
| 45 | 10 | 8.33±0.16 | 8.90±0.18 | 8.58±0.35 | 9.28±0.16 |
|  | 30 | 10.03±0.19 | 11.32±0.08 | 10.79±0.36 | 11.60±0.08 |
|  | 60 | 10.66±0.08 | 12.04±0.12 | 11.19±0.24 | 12.30±0.40 |
|  | 240 | 12.45±0.08 | 13.87±0.15 | 12.77±0.24 | 14.25±0.35 |
|  | 480 | 13.74±0.32 | 14.78±0.19 | 14.31±0.42 | 15.22±0.12 |


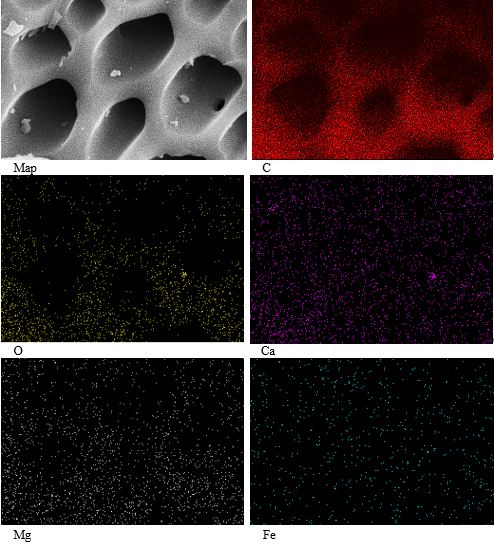


Fig. S1 The elements distribution maps of bush biochar (BB)


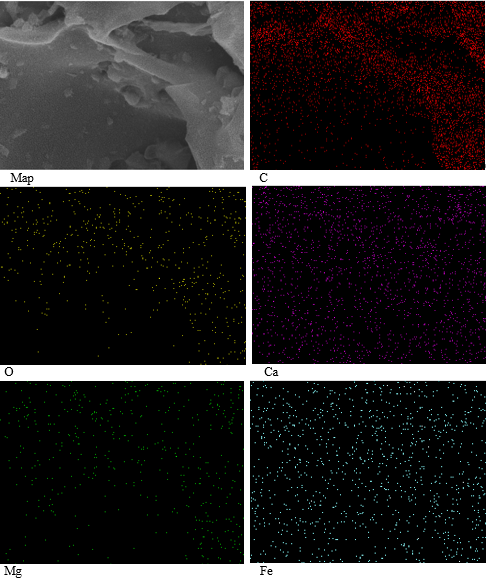


Fig. S2 The elements distribution maps of bush biochar (PB)


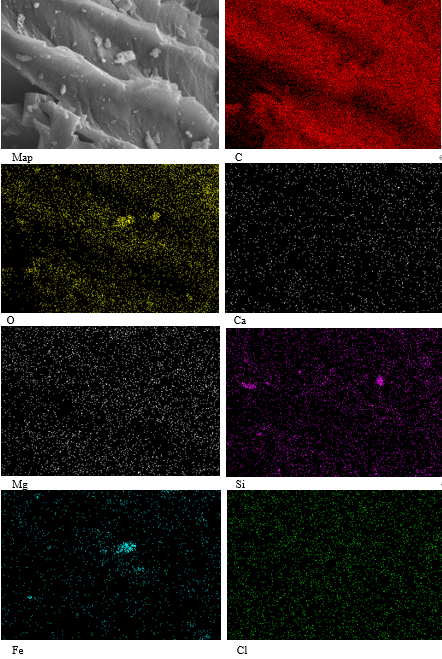


Fig. S3 The elements distribution maps of aged bush biochar (ABB) sorbed 160 mg L^-1^ 2,4,6-TCP


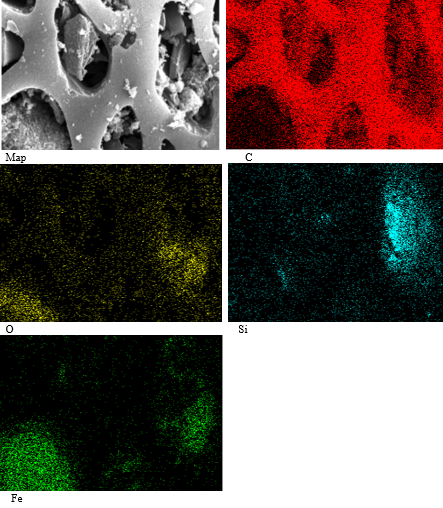


Fig. S4 The elements distribution maps of aged peanut biochar (APB) sorbed 160 mg L^-1^ 2,4,6-TCP
